# Physical Activity and Sedentary Time in Korean Adults before and during the COVID-19 Pandemic Using Data from the Korea National Health and Nutritional Examination Survey

**DOI:** 10.3390/jpm12081217

**Published:** 2022-07-26

**Authors:** So Young Kim, Dae Myoung Yoo, Mi Jung Kwon, Ji Hee Kim, Joo-Hee Kim, Woo Jin Bang, Hyo Geun Choi

**Affiliations:** 1 Bundang CHA Medical Center, Department of Otorhinolaryngology-Head and Neck Surgery, CHA University, Seongnam 13488, Korea; sossi81@hanmail.net; 2 Hallym Data Science Laboratory, Hallym University College of Medicine, Anyang 14066, Korea; ydm1285@naver.com; 3 Department of Pathology, Hallym Sacred Heart Hospital, Hallym University College of Medicine, Anyang 14068, Korea; mulank@hanmail.net; 4 Department of Neurosurgery, Hallym University College of Medicine, Anyang 14068, Korea; kimjihee.ns@gmail.com; 5 Department of Medicine, Division of Pulmonary, Allergy, and Critical Care Medicine, Hallym Sacred Heart Hospital, Hallym University College of Medicine, Anyang 14068, Korea; luxjhee@gmail.com; 6 Department of Urology, Hallym Sacred Heart Hospital, Hallym University College of Medicine, Anyang 14068, Korea; 7 Department of Otorhinolaryngology-Head and Neck Surgery, Hallym University College of Medicine, Anyang 14068, Korea

**Keywords:** physical activity, COVID-19, risk factors, cohort studies, epidemiology

## Abstract

Several recent studies suggested reduced physical activity (PA) related to the COVID-19 pandemic without consensus. This study investigated the changes in PA and sedentary time during the COVID-19 pandemic compared to before the COVID-19 era. The Korea National Health and Nutrition Examination Survey 2019 and 2020 were used. The ≥19-year-old population was examined in 2019 and 2020 for time spent engaging in high- and moderate-intensity PA and sedentary time. Based on the recommended level of PA by the World Health Organization, ≥75 min/wk of high PA and ≥150 min/of moderate PA were classified. A sedentary time of ≥120 min/d was selected. Multiple logistic regression analysis with complex sampling was conducted for ≥75 min/wk of high PA, ≥150 min/wk of moderate PA, and ≥120 min/d of sedentary time in the 2020 group compared to the 2019 group. The ≥75 min/wk high PA was not lower in the 2020 group than in the 2019 group (adjusted odds ratio (aOR) = 0.96, 95% confidence intervals (95% CIs) = 0.79–1.18, P = 0.696). The ≥150 min/wk of moderate PA was also not lower in the 2020 group (aOR = 1.12, 95% CI = 0.94–1.32, *p* = 0.201). However, the ≥150 min/wk of moderate PA in the female group was higher in the 2020 group than in the 2019 group (aOR = 1.29, 95% CI = 1.01–1.65, *p* = 0.042). Sedentary time ≥ 120 min/d was lower in the 2020 group (aOR = 0.35, 95% CI = 0.17–0.72, *p* = 0.005). This association was consistent in the 19–39-year-old and female group. In conclusion, during the COVID-19 pandemic, high and moderate PA were not decreased in Korean adults, while sedentary time was reduced.

## 1. Introduction

Physical activity (PA) is one of the essential parts of daily life to maintain physical and mental health. PA is known to protect against many noncommunicable diseases, including cardiovascular and respiratory diseases [1,2]. In addition to preventing noncommunicable diseases, PA is supposed to relieve the disease burden of communicable diseases. PA has been acknowledged to promote homeostasis of the immune system and alleviate circulatory inflammatory responses and sarcopenia [3]. In the general population, PA can reduce the risk of social isolation and psychological illness, such as depression and anxiety [3,4]. In addition, sedentary behavior was reported to be associated with lower health-related quality of life and increased risk of chronic diseases [5,6]. 

The favorable effects of PA have also been proposed to prevent COVID-19 contraction. It was reported that increased PA was associated with a lower risk of COVID-19 infection (odds ratio (OR) = 0.80, 95% CI = 0.69–0.93) [7]. Thus, although the social distancing policy and the lockdown act limited PA during the COVID-19 pandemic, regular PA and home training are recommended to alleviate the risk of COVID-19 [8].

However, a number of recent studies described the adverse impacts of the COVID-19 pandemic on PA [9,10,11]. The direct effects of COVID-19 on physical health could impair PA. In addition to active patients with COVID-19, the long-term persistent symptoms of COVID-19 could limit PA in patients suffering from COVID-19 [10]. Compared to pre-COVID-19, walking time was significantly decreased six months after COVID-19 (60 min vs. 120 min, *p* < 0.05) [10]. In addition, the quarantine measures restricted PA in the general population [9]. During the lockdown period, PA was reduced, and it had adverse impacts on physical health and morbidities, such as increased body mass and neurologic diseases [11]. Sedentary behavior was also reported to be increased during the early COVID-19 pandemic period in U.S. children [12]. Moreover, the participants who spent more time sedentary demonstrated higher risk of depressive symptoms during the COVID-19 pandemic [13].

Although many prior studies pointed to reduced PA during the COVID-19 pandemic, to our knowledge, there has been little knowledge on the age- and sex-specific impacts on PA. Therefore, we questioned the impact of the COVID-19 pandemic on PA and sedentary behavior. To address this research question, this study investigated the difference in PA and sedentary time before and during the COVID-19 pandemic era in an adult population. Using subgroup analysis, age- and sex-specific features for PA and sedentary time were examined. We used a nationwide cohort population that represents Korean adults and minimizes potential selection bias. In addition, the level of PA was surveyed using the global recommendations on PA for health from the World Health Organization in 2010, which were declared as ≥75 min/wk of vigorous PA or ≥150 min/wk of moderate PA.

## 2. Materials and Methods

### 2.1. Study Population and Data Collection

This cross-sectional study was exempt from institutional ethical review from the Institutional Review Board based on the bioethics law of South Korea, which indicated the exemption of the ethical review for the study conducted by the South Korean national government to improve public health. All Korea National Health and Nutrition Examination survey (KNHANES) data were analyzed under the regulations of the Institutional Review Board of the Centers for Disease Control and Prevention of Korea (KCDC) [14]. All study participants were civilians of South Korea. The current study complied with the STROBE guideline.

The 8th KNHANES from 2019 and 2020 was analyzed. The 2019 group was surveyed from 1 January 2019 through 31 December 2019 and the 2020 group was surveyed from 1 January 2020 through 31 December 2020. From 1 January 2020 through 31 December, the social distancing strategies were enacted based on the basic guidelines for distancing in daily life in Korea [15]. KNHANES is a population-based cross-sectional survey to evaluate health-related behavior, health condition, and nutritional state. To sample the representative population of Korea, the KNHANES selected a statistically sampled population and applied sample weights [14,16].

### 2.2. Survey

#### 2.2.1. Exposure

In 2019 and 2020, adult participants were enrolled. The participants in 2019 were not identical. The 2020 participants were newly enrolled.

#### 2.2.2. Outcome

Participants were asked their histories of the intensity of PA and sedentary behavior using the Korean version of the modified Global Physical Activity Questionnaire whose validity was verified in a previous study [17,18,19]. Regarding high PA, participants were asked the following questions: “On how many days in the typical week and how much time a day did you do the work-related, high-intensity physical activity at least 10 min at once?”, “On how many days in the typical week and how much time a day did you do the high-intensity sports, exercise, or leisure time activity at least 10 min at once?”. If participants did ≥75 min per week of high PA from the sum of work and leisure time activity, they were classified as ‘≥75 min/wk of high PA’, and others were classified as ‘<75 min/wk of high PA’.

Regarding moderate-intensity PA (moderate PA), participants were asked the following questions: “On how many days in the typical week and how much time a day did you do the work-related, moderate-intensity physical activity at least 10 min at once?”, “On how many days in the typical week and how much time a day did you do the moderate-intensity sports, exercise, or leisure time activity at least 10 min at once?”. If participants did ≥150 min per week of moderate PA from the sum of work and leisure time activity, they were classified as ‘≥150 min/wk of moderate PA’, and others were classified as ‘<150 min/wk of moderate PA’.

Regarding sedentary time, participants were asked the following question: “How much time a day do you usually sit or lie down?”. If participants did ≥120 min per day of sedentary time, they were classified as ‘≥120 min/d of sedentary time’, and others were classified as ‘<120 min/d of sedentary time’.

#### 2.2.3. Covariate

Income was recalculated by dividing total household income by the square root of the number of household members [20]. The level of income was classified into 5 groups. Employment status was classified as unemployed or employed. Educational status was divided into elementary school or under, unknown; junior high school; high school; college or over. The type of house was surveyed as detached house, condominium, townhouse, and others. Marriage status was divided into married, unmarried, and unknown. BMI (kg/m^2^) was calculated using height and weight. Smoking status (nonsmoker, past smoker, and current smoker) and alcohol consumption (nonconsumer, 1 to 5 times/month, ≥2 times/week) were surveyed. Sleep time was calculated as 5/7 times on weekdays plus 2/7 times on weekends [21]. The past medical histories were collected by asking whether participants had received a doctor diagnosis of each disease.

### 2.3. Statistical Analysis

The demographics and variables of 2019 and 2020 were analyzed using linear regression analysis with complex sampling and the chi-square test with Rao–Scott correction to represent the entire population [14].

The odds ratios (ORs) for ≥75 min/wk of high PA, ≥150 min/wk of moderate PA, and ≥120 min/d of sedentary time in 2020 compared to 2019 were analyzed using multiple logistic regression analysis with complex sampling. Crude and adjusted (continuous: age, income, BMI, and sleep time; categorical: sex, employment, education, house type, marital status, smoking, alcohol consumption, hypertension, dyslipidemia, stroke, ischemic heart disease, osteoarthritis, rheumatoid arthritis, diabetes mellitus, chronic kidney disease, and gout) models were designed. Subgroup analyses by age and sex were analyzed.

In addition, we analyzed ORs for ≥300 min/wk of PA at work and for ≥300 min/wk of PA in leisure time in 2020 compared to 2019 with subgroup analyses according to age and sex. Both at work and in leisure time, high PAs were calculated to be double due to the higher intensity compared to moderate PA (Tables S1 and S2).

A *p* value < 0.05 indicated statistical significance. The 95% confidence intervals (CIs) were included. The weights recommended by the KNHANES were applied. SPSS ver. 25.0 (IBM, Armonk, NY, USA) was used for analyses.

## 3. Results

**Figure 1:**
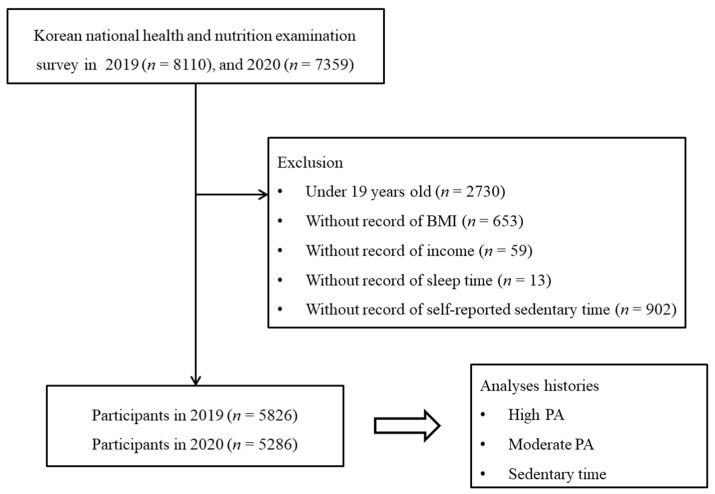
Study design of the present study. From the Korean National Health and Nutritional Examination data, a total of 5826 individuals in the 2019 cohort were compared with 5286 individuals in the 2020 cohort for high-intensity physical activity (PA), moderate PA, and sedentary time.

Of the 15,469 total participants (8110 in 2019; 7359 in 2020), the following were excluded from this study: participants under 19 years old (*n* = 2730), without records of body mass index (BMI, *n* = 653), income (*n* = 59), sleep time (*n* = 13), and self-reported sedentary time (*n* = 902). Finally, 11,112 participants (5826 in 2019; 5286 in 2020) 19 through 80+ years old were included in this study (Figure 1). Then, we analyzed the histories of the intensity of physical activity (PA) and sedentary time between 2019 and 2020.

The rates of time spent on high PA and moderate PA in the 2019 and 2020 groups were not different (Table 1). A total of 9.1% of the 2019 group and 8.9% of the 2020 group reported ≥75 min/wk of high PA (*p* = 0.814). For moderate PA, 14.9% of the 2019 group and 16.7% of the 2020 group described ≥150 min/wk of moderate PA (*p* = 0.097). Sedentary time was not significantly different between the 2020 group and the 2019 group (*p* = 0.071). 

The odds for ≥75 min/wk of high PA were not decreased in the 2020 group compared to the 2019 group (adjusted OR (aOR) = 0.96, 95% CI = 0.79–1.18, *p* = 0.696, Table 2). The results on the association of ≥75 min/wk of high PA with the 2020 group were consistent in all age and sex subgroups (all *p* > 0.05).

The odds for moderate PA ≥ 150 min/wk were not decreased in the 2020 group compared to the 2019 group (aOR = 1.12, 95% CI = 0.94–1.32, *p* = 0.201, Table 3). However, in the female group, the odds for moderate PA ≥ 150 min/wk were 1.29 times higher in the 2020 group than in the 2019 group (aOR = 1.29, 95% CI = 1.01–1.65, *p* = 0.042). All other age and sex subgroups did not show an association of ≥ 150 min/wk of moderate PA with the 2020 group (all *p* > 0.05).

The ≥120 min/d sedentary time was 0.35 times lower in the 2020 group than in the 2019 group (95% CI = 0.17–0.72, *p* = 0.005, Table 4). The 19–39-year-old group demonstrated 0.13 times lower odds for the ≥120 min/d of sedentary time in the 2020 group than the 2019 group (95% CI = 0.02–0.89, *p* = 0.038). In addition, the female group indicated 0.74 times lower odds for ≥ 120 min/d of sedentary time in the 2020 group than in the 2019 group (95% CI = 0.07–0.53, *p* = 0.001).

The odds for ≥300 min/wk of PA at work or leisure were not different between the 2019 and 2020 groups (Tables S1 and S2).

The rates of high PA, moderate PA, and sedentary time were compared between male and female groups (Table 5 and Table 6). Both 2019 and 2020 groups demonstrated a lower rate of high PA and moderate PA in women. Sedentary time was lower in females than males only in the 2019 group (*p* = 0.026, Table 6).

## 4. Discussion

Moderate- or high-intensity PA was not different before and during the COVID-19 pandemic in the overall adult Korean cohort. However, moderate PA was higher during the COVID-19 pandemic in the female group. Sedentary time was lower during the COVID-19 pandemic. In particular, the young adult and female groups demonstrated lower sedentary time during the COVID-19 pandemic.

A number of recent studies have reported the impact of the COVID-19 pandemic on PA [11,22].

In Spain, ≥75 min/wk of high PA was decreased by 10.7% in adults [22]. The strict confinement strategies from the government could have reduced high PA during the COVID-19 lockdown period in this population. On the other hand, ≥150 min/wk of moderate PA showed little change during the COVID-19 pandemic in the same population (1.4%) [22]. The different quarantine strategies in Korea without a complete lockdown period could have resulted in no significant decrease in high or moderate PA in Korean adults. An online survey study in Canada demonstrated multidirectional changes in PA during the COVID-19 pandemic [23]. During the COVID-19 pandemic, 40.5% of inactive participants became inactive, while 40.3% of active participants became more active [23]. Thus, it can be presumed that personal characteristics could impact changes in PA during the COVID-19 pandemic.

The moderate PA was high during the COVID-19 pandemic in the women’s group only. The sex difference in PA during the COVID-19 pandemic has been suggested in another previous report [24]. Women were reported to be more well adapted and maintained PA during the lockdown period compared to men [24]. As previous researchers have suggested, sex differences in coping with and adapting to the pandemic crisis could have induced a specific increase in moderate PA in women in this study. Moreover, the higher PA in women during the COVID-19 pandemic can be attributed to the increased time spent with family and on household chores. An online survey study reported that 34.3% of working women experienced a higher physical load owing to household chores during the COVID-19 lockdown [25]. Another online study demonstrated that women were more dedicated to home care and domestic chores during the COVID-19 lockdown [26].

Sedentary time was lower during the COVID-19 pandemic in the present study. In particular, the young adult and female groups demonstrated lower sedentary time than the 2020 group in this study. The current results are in conflict with the findings of previous studies describing decreased sitting time during the COVID-19 pandemic [12,27]. The differences in quarantine measures and social distancing strategies could have influenced these conflicting results. A review study indicated that age and gender are some of the main determining factors for PA during the COVID-19 pandemic [9]. The increased physical load from domestic chored and child care could have mediated the decreased sedentary time in women because of the lockdown of schools and workplaces during the COVID-19 pandemic period. In addition, home-based exercise could have compensated for outdoor PA during the COVID-19 pandemic [28,29]. As the young adult population may have more access to mobile devices and media content than the older population, accessibility of home-based exercise programs may have been easier.

This study was based on a representative nationwide population cohort. The survey questionnaires were based on the WHO recommendation and regularly validated by a statistician in the Korean government. This study compared before and during the COVID-19 pandemic based on an independent cohort population. 

However, a few limitations should be considered when interpreting the current findings. As each cohort was newly selected and examined in 2019 and 2020, follow-up data were lacking in the present study. Due to the cross-sectional study design, the causality between PA and the COVID-19 pandemic cannot be evaluated. In addition, regional or ethnic differences in COVID-19 should be considered [30,31]. The infection rate of COVID-19 was relatively low in Korea compared to Europe and other countries during 2020. As the Korean government did not completely lock down during the COVID-19 pandemic, social activities and PA may have been much more feasible than in other countries. The Asian gender roles are also influencing factors for the women-specific increase in moderate PA and decrease in sedentary time in the present study. We cannot use the WHO international terminology for PA, because this study classified the PA based on the survey questionnaire without measuring heart rate. Moreover, although many variables were adjusted in the current study, some potential confounders, such as types of job and the individual characteristics of introvert or extrovert, could not be considered. Lastly, this study examined the early pandemic era, and the long-term effects of the COVID-19 pandemic on PA warrant further study.

## 5. Conclusions

High and moderate PA were not lower during the COVID-19 pandemic in Korean adults. In the female population, moderate PA was higher during the COVID-19 pandemic. Sedentary time was lower during the COVID-19 pandemic in Korean adults, which was remarkable in the young adult and female groups.

## Figures and Tables

**Table 1:** General characteristics of participants.

Characteristics		Year		*p*-Value *
		2019	2020	
Age (yrs, mean, SD)		51.3 (16.7)	51.0 (16.9)	0.551
Age groups (yrs, *n*, %)				0.942
	19–39	1596 (27.4)	1497 (28.3)	
	40–59	2190 (37.6)	1913 (36.2)	
	≥60	2040 (35.0)	1876 (35.5)	
Sex (*n*, %)				0.400
	Males	2587 (49.7)	2398 (45.4)	
	Females	3239 (50.3)	2888 (54.6)	
Income (mean, SD)		3.2 (1.4)	3.3 (1.3)	0.446
Income group (*n*, %)				0.861
	1 (lowest)	841 (10.8)	662 (10.0)	
	2	1056 (16.1)	931 (15.1)	
	3	1178 (21.4)	1110 (21.6)	
	4	1343 (25.1)	1265 (25.5)	
	5 (highest)	1408 (26.6)	1318 (27.9)	
Employment (*n*, %)				0.356
	Unemployed	2266 (34.9)	2073 (36.1)	
	Employed	3560 (65.1)	3213 (63.9)	
Educational status (*n*, %)				0.281
	Elementary school or under, unknown	1038 (12.2)	850 (10.7)	
	Junior high school	549 (7.7)	512 (7.6)	
	High school	1961 (35.8)	1878 (38.9)	
	College or over	2278 (44.2)	2046 (42.8)	
House type (*n*, %)				0.710
	Detached house	1777 (27.4)	1676 (27.3)	
	Condominium	3216 (57.3)	2964 (64.3)	
	Raw houses	784 (14.5)	618 (11.9)	
	Others	49 (9.8)	28 (8.4)	
Marriage status (*n*, %)				0.049 *
	Married	4017 (66.7)	3459 (63.3)	
	Unmarried	783 (13.0)	709 (19.0)	
	Unknown	1026 (23.3)	1118 (26.7)	
Body mass index (mean, SD)		23.9 (3.6)	24.2 (3.8)	<0.001 *
Smoking status (*n*, %)				0.521
	Nonsmoker	3465 (56.6)	3160 (56.5)	
	Past smoker	1346 (23.1)	1231 (24.1)	
	Current smoker	1015 (20.3)	895 (19.4)	
Alcohol consumption (*n*, %)				0.115
	Nonconsumer	2660 (41.8)	2541 (43.7)	
	1 to 5 times/mo	1931 (36.4)	1667 (33.8)	
	≥2 times/week	1235 (21.8)	1078 (22.4)	
Sleep duration (mean, SD)		6.8 (1.3)	6.8 (1.4)	0.874
Hypertension (*n*, %)		1449 (20.2)	1288 (19.7)	0.691
Dyslipidemia (*n*, %)		1137 (16.6)	1155 (18.0)	0.117
Stroke (*n*, %)		137 (1.9)	114 (1.6)	0.242
Ischemic heart disease (*n*, %)		179 (2.5)	173 (2.2)	0.458
Osteoarthritis (*n*, %)		661 (8.4)	599 (8.2)	0.813
Rheumatoid arthritis (*n*, %)		117 (1.8)	91 (1.2)	0.031 *
Diabetes mellitus (*n*, %)		559 (7.8)	557 (8.5)	0.341
Chronic kidney disease (*n*, %)		24 (0.4)	99 (1.6)	<0.001 *
Gout (*n*, %)		102 (2.0)	100 (1.9)	0.694
High PA (*n*, %)				0.814
	≥75 min/wk	438 (9.1)	396 (8.9)	
	<75 min/wk	5388 (90.9)	4890 (91.1)	
Moderate PA (*n*, %)				0.097
	≥150 min/wk	818 (14.9)	837 (16.7)	
	<150 min/wk	5008 (85.1)	4449 (83.3)	
Sedentary time (*n*, %)				0.071
	≥240 min/d	21 (0.3)	39 (0.7)	
	≥120 min/d and <240 min/d	482 (7.9)	394 (7.5)	
	<120 min/d	5323 (91.8)	4853 (91.8)	

SD: standard deviation, PA: physical activity. * The general characteristics in 2019 and 2020 were compared using linear regression analysis with complex sampling for continuous variables, and chi-square test with Rao-Scott correction for categorical variables. Significance at *p* < 0.05.

**Table 2:** Odds ratios (95% confidence intervals) for ≥75 min/wk of high-intensity physical activity in 2020 compared to 2019 with subgroup analyses according to age and sex.

Characteristics		Odds Ratios for ≥75 min/wk of High PA in 2020 Compared to 2019			
		Crude	*p*-Value *	Adjusted ^†^	*p*-Value *
Total participants (*n* = 11,112)		0.98 (0.80–1.20)	0.814	0.96 (0.79–1.18)	0.696
Age					
	19–39 years old (*n* = 3093)	0.93 (0.72–1.21)	0.586	0.93 (0.71–1.23)	0.629
	40–59 years old (*n* = 4103)	1.00 (0.76–1.32)	0.983	1.03 (0.77–1.37)	0.860
	≥60 years old (*n* = 3916)	1.11 (0.71–1.73)	0.649	1.03 (0.66–1.60)	0.913
Sex					
	Males (*n* = 4985)	0.88 (0.70–1.11)	0.270	0.87 (0.69–1.10)	0.241
	Females (*n* = 6127)	1.22 (0.90–1.66)	0.191	1.21 (0.89–1.64)	0.218

Abbreviations: BMI, body mass index; high PA, high-intensity physical activity. * Logistic regression, significance at *p* < 0.05. ^†^ Adjusted for age, sex, income, employment, educational status, house type, marriage status, BMI, smoking status, alcohol consumption, sleep duration, hypertension, dyslipidemia, stroke, ischemic heart disease, osteoarthritis, rheumatoid arthritis, diabetes mellitus, chronic kidney disease, and gout.

**Table 3:** Odds ratios (95% confidence intervals) for ≥150 min/wk of moderate-intensity physical activity in 2020 compared to 2019 with subgroup analyses according to age and sex.

Characteristics		Odds Ratios for ≥150 min/wk of Moderate PA in 2020 Compared to 2019			
		Crude	*p*-Value *	Adjusted ^†^	*p*-Value *
Total participants (*n* = 11,112)		1.15 (0.97–1.36)	0.100	1.12 (0.94–1.32)	0.201
Age					
	19–39 years old (*n* = 3093)	1.21 (0.97–1.51)	0.084	1.23 (0.97–1.55)	0.085
	40–59 years old (*n* = 4103)	1.01 (0.79–1.28)	0.971	1.01 (0.79–1.28)	0.971
	≥60 years old (*n* = 3916)	1.33 (1.00–1.77)	0.050	1.23 (0.92–1.66)	0.160
Sex					
	Males (*n* = 4985)	1.02 (0.85–1.24)	0.813	1.01 (0.84–1.22)	0.915
	Females (*n* = 6127)	1.36 (1.07–1.74)	0.013 *	1.29 (1.01–1.65)	0.042 *

Abbreviations: BMI, body mass index; moderate PA, moderate-intensity physical activity. * Logistic regression, significance at *p* < 0.05. ^†^ Adjusted for age, sex, income, employment, educational status, house type, marriage status, BMI, smoking status, alcohol consumption, sleep duration, hypertension, dyslipidemia, stroke, ischemic heart disease, osteoarthritis, rheumatoid arthritis, diabetes mellitus, chronic kidney disease, and gout.

**Table 4:** Odds ratios (95% confidence intervals) for ≥ 120 min/d of sedentary time in 2020 compared to 2019 with subgroup analyses according to age and sex.

Characteristics		Odds Ratios for ≥120 min/d of Sedentary Time in 2020 Compared to 2019			
		Crude	*p*-Value *	Adjusted ^†^	*p*-Value *
Total participants (*n* = 11,112)		0.39 (0.18–0.84)	0.017 *	0.35 (0.17–0.72)	0.005 *
Age					
	19–39 years old (*n* = 3093)	0.18 (0.03–0.96)	0.045 *	0.13 (0.02–0.89)	0.038 *
	40–59 years old (*n* = 4103)	0.35 (0.12–1.04)	0.059	0.35 (0.11–1.08)	0.067
	≥60 years old (*n* = 3916)	0.59 (0.26–1.34)	0.203	0.52 (0.23–1.21)	0.131
Sex					
	Males (*n* = 4985)	0.67 (0.23–1.96)	0.461	0.74 (0.26–2.14)	0.582
	Females (*n* = 6127)	0.24 (0.09–0.65)	0.005 *	0.20 (0.07–0.53)	0.001 *

Abbreviations: BMI, body mass index. * Logistic regression, significance at *p* < 0.05. ^†^ Adjusted for age, sex, income, employment, educational status, house type, marriage status, BMI, smoking status, alcohol consumption, sleep duration, hypertension, dyslipidemia, stroke, ischemic heart disease, osteoarthritis, rheumatoid arthritis, diabetes mellitus, chronic kidney disease, and gout.

**Table 5:** General characteristics of participants.

Characteristics		Sex		*p*-Value *
		Males	Females	
High PA (*n*, %)				<0.001 *
	≥75 min/wk	298 (13.7)	140 (4.6)	
	<75 min/wk	2289 (86.3)	3099 (95.4)	
Moderate PA (*n*, %)				<0.001 *
	≥150 min/wk	484 (19.5)	334 (10.3)	
	<150 min/wk	2103 (80.5)	2905 (89.7)	
Sedentary time (*n*, %)				0.325
	≥240 min/d	2378 (92.3)	2945 (91.2)	
	≥120 min/d and <240 min/d	196 (7.4)	286 (8.5)	
	<120 min/d	13 (0.3)	08 (0.2)	

SD: standard deviation, PA: physical activity. * The general characteristics of males and females examined in 2019 were compared using linear regression analysis with complex sampling for continuous variables, and chi-square test with Rao–Scott correction for categorical variables. Significance at *p* < 0.05.

**Table 6:** General characteristics of participants.

Characteristics		Sex		*p*-Value *
		Males	Females	
High PA (*n*, %)				<0.001 *
	≥75 min/wk	259 (12.2)	137 (5.5)	
	<75 min/wk	2139 (87.8)	2751 (94.5)	
Moderate PA (*n*, %)				<0.001 *
	≥150 min/wk	475 (19.8)	362 (13.6)	
	<150 min/wk	1923 (80.2)	2526 (86.4)	
Sedentary time (*n*, %)				0.026 *
	≥240 min/d	2183 (91.8)	2670 (91.8)	
	≥120 min/d and <240 min/d	204 (7.9)	190 (7.5)	
	<120 min/d	11 (0.3)	28 (0.7)	

SD: standard deviation, PA: physical activity. * The general characteristics of males and females examined in 2020 were compared using linear regression analysis with complex sampling for continuous variables, and chi-square test with Rao-Scott correction for categorical variables. Significance at *p* < 0.05.

## Data Availability

Restrictions apply to the availability of these data. Data were obtained from the Korea National Health and Nutrition Examination Survey and are available at https://knhanes.kdca.go.kr/knhanes/main.do (accessed on 20 January 2022).

## Supplementary Materials

The following are available online at https://www.mdpi.com/article/10.3390/jpm12081217/s1, Table S1: Odds ratios (95% confidence intervals) for ≥300 min/wk of physical activity at work in 2020 compared to 2019 with subgroup analyses according to age and sex; Table S2: Odds ratios (95% confidence intervals) for physical activity at leisure time in 2020 compared to 2019 with subgroup analyses according to age and sex.
