# Supplementary material for: Physical Activity and Sedentary Time in Korean Adults before and during the COVID-19 Pandemic Using Data from the Korea National Health and Nutritional Examination Survey
Source: J Pers Med. 2022 Jul 26;12(8):1217. doi: 10.3390/jpm12081217 (PMC9394236; doi:10.3390/jpm12081217)
Supplement: Supplementary file 1 [file jpm-12-01217-s001.zip › jpm-1777550-supplementary.pdf]

Journal of *Personalized Medicine*

Supplementary Materials

Physical Activity and Sedentary Time in Korean Adults before and during the COVID-19 Pandemic Using Data from the Korea National Health and Nutritional Examination Survey

So Young Kim [1], Dae Myoung Yoo [2], Mi Jung Kwon [3], Ji Hee Kim [4], Joo-Hee Kim [5], Woo Jin Bang [6,*] and Hyo Geun Choi [1,7,*]

1 Bundang CHA Medical Center, Department of Otorhinolaryngology-Head and Neck Surgery, CHA University, Seongnam 13488, Korea; sossi81@hanmail.net
2 Hallym Data Science Laboratory, Hallym University College of Medicine, Anyang 14066, Korea; ydm1285@naver.com
3 Department of Pathology, Hallym Sacred Heart Hospital, Hallym University College of Medicine, Anyang 14068, Korea; mulank@hanmail.net
4 Department of Neurosurgery, Hallym University College of Medicine, Anyang 14068, Korea; kimjihee.ns@gmail.com
5 Department of Medicine, Division of Pulmonary, Allergy, and Critical Care Medicine, Hallym Sacred Heart Hospital, Hallym University College of Medicine, Anyang 14068, Korea; luxjhee@gmail.com
6 Department of Urology, Hallym Sacred Heart Hospital, Hallym University College of Medicine, Anyang 14068, Korea
7 Department of Otorhinolaryngology-Head and Neck Surgery, Hallym University College of Medicine, Anyang 14068, Korea
* Correspondence: yybbang@gmail.com (W.J.B.); pupen@naver.com (H.G.C.)

Table S1. Odds ratios (95% confidence intervals) for ≥ 300 min/wk of physical activity at work in 2020 compared to 2019 with subgroup analyses according to age and sex.

Characteristics	Odds Ratios for ≥ 300 min/wk of PA at Work in 2020 Compared to 2019			
	Crude	*p*-Value *	Adjusted †	*p*-Value *
Total participants (*n* = 11,112)	1.19 (0.88–1.60)	0.258	1.21 (0.90–1.64)	0.213
Age				
19–39 years old (*n* = 3093)	1.33 (0.89–1.98)	0.166	1.40 (0.93–2.10)	0.106
40–59 years old (*n* = 4103)	1.06 (0.69–1.64)	0.778	1.10 (0.71–1.71)	0.674
≥ 60 years old (*n* = 3916)	0.96 (0.50–1.87)	0.911	0.92 (0.47–1.80)	0.800
Sex				
Males (*n* = 4985)	1.08 (0.78–1.50)	0.629	1.08 (0.77–1.50)	0.659
Females (*n* = 6127)	1.48 (0.88–2.49)	0.140	1.60 (0.95–2.71)	0.078

Abbreviations: BMI, body mass index; PA, physical activity. * Logistic regression, Significance at $p < 0.05$. † Adjusted for age, sex, income, employment, educational status, house type, marriage status, BMI, smoking status, alcohol consumption, sleep duration, hypertension, dyslipidemia, stroke, ischemic heart disease, osteoarthritis, rheumatoid arthritis, diabetes mellitus, chronic kidney disease, and gout.

Table S2. Odds ratios (95% confidence intervals) for physical activity at leisure time in 2020 compared to 2019 with subgroup analyses according to age and sex.

**Characteristics**	**Odds Ratios for ≥ 300 min/wk of PA at Leisure Time in 2020 Compared to 2019**			
	**Crude**	***p*-Value ***	**Adjusted †**	***p*-Value ***
Total participants (*n* = 11,112)	1.08 (0.87–1.33)	0.504	1.03 (0.83–1.27)	0.806
Age				
19–39 years old (*n* = 3093)	0.99 (0.76–1.29)	0.943	0.96 (0.73–1.26)	0.756
40–59 years old (*n* = 4103)	1.12 (0.83–1.52)	0.446	1.15 (0.85–1.57)	0.364
≥ 60 years old (*n* = 3916)	1.22 (0.83–1.80)	0.311	1.14 (0.77–1.69)	0.514
Sex				
Males (*n* = 4985)	0.99 (0.79–1.26)	0.963	0.97 (0.77–1.23)	0.804
Females (*n* = 6127)	1.24 (0.91–1.69)	0.167	1.16 (0.86–1.58)	0.337

Abbreviations: BMI, body mass index; PA, physical activity. * Logistic regression, Significance at $p < 0.05$. † Adjusted for age, sex, income, employment, educational status, house type, marriage status, BMI, smoking status, alcohol consumption, sleep duration, hypertension, dyslipidemia, stroke, ischemic heart disease, osteoarthritis, rheumatoid arthritis, diabetes mellitus, chronic kidney disease, and gout.
